# Stromal CCL5 Promotes Breast Cancer Progression by Interacting with CCR3 in Tumor Cells

**DOI:** 10.3390/ijms22041918

**Published:** 2021-02-15

**Authors:** Mio Yamaguchi, Kiyoshi Takagi, Koki Narita, Yasuhiro Miki, Yoshiaki Onodera, Minoru Miyashita, Hironobu Sasano, Takashi Suzuki

**Affiliations:** 1 Department of Pathology and Histotechnology, Graduate School of Medicine, Tohoku University, 2-1 Seiryo-machi, Aoba-ku, Sendai 980-8575, Miyagi, Japan; mio.yamaguchi.p3@dc.tohoku.ac.jp (M.Y.); koki.narita.s7@dc.tohoku.ac.jp (K.N.); t-suzuki@patholo2.med.tohoku.ac.jp (T.S.); 2 Department of Disaster Obstetrics and Gynecology, International Research Institute of Disaster Science, Tohoku University, Sendai 980-8574, Miyagi, Japan; miki@patholo2.med.tohoku.ac.jp; 3 Department of Anatomic Pathology, Graduate School of Medicine, Tohoku University, Sendai 980-8575, Miyagi, Japan; golgo04@magic.odn.ne.jp (Y.O.); hsasano@patholo2.med.tohoku.ac.jp (H.S.); 4 Department of Breast and Endocrine Surgical Oncology, Graduate School of Medicine, Tohoku University, Sendai 980-8575, Miyagi, Japan; atihsayim8m8@med.tohoku.ac.jp; 5 Department of Pathology, Tohoku University Hospital, Sendai 980-8574, Miyagi, Japan

**Keywords:** breast carcinoma, chemokine, immunohistochemistry, prognosis, tumor microenvironment

## Abstract

Chemokines secreted from stromal cells have important roles for interactions with carcinoma cells and regulating tumor progression. C-C motif chemokine ligand (CCL) 5 is expressed in various types of stromal cells and associated with tumor progression, interacting with C-C chemokine receptor (CCR) 1, 3 and 5 expressed in tumor cells. However, the expression on CCL5 and its receptors have so far not been well-examined in human breast carcinoma tissues. We therefore immunolocalized CCL5, as well as CCR1, 3 and 5, in 111 human breast carcinoma tissues and correlated them with clinicopathological characteristics. Stromal CCL5 immunoreactivity was significantly correlated with the aggressive phenotype of breast carcinomas. Importantly, this tendency was observed especially in the CCR3-positive group. Furthermore, the risk of recurrence was significantly higher in the patients with breast carcinomas positive for CCL5 and CCR3 but negative for CCR1 and CCR5, as compared with other patients. In summary, the CCL5-CCR3 axis might contribute to a worse prognosis in breast cancer patients, and these findings will contribute to a better understanding of the significance of the CCL5/CCRs axis in breast carcinoma microenvironment.

## 1. Introduction

Breast cancer is one of the most common types of malignant neoplasms in women worldwide [1]. Although the endocrine therapy targeting estrogen signaling pathway and cytotoxic chemotherapy are widely used and have remarkably improved the prognosis of breast cancer patients, a de novo and/or acquired resistance to these treatments are often experienced [2], and about 25% of breast cancer patients who receive adjuvant chemotherapy are reported to recur after the therapy [3]. Therefore, it is required to discover new therapeutic targets for breast cancer to improve the clinical outcome of patients.

The tumor microenvironment is composed of not only carcinoma cells but various stromal cells, including macrophages, leukocytes, fibroblasts and platelets. Stromal cells in tumor tissues regulate tumor progression via mainly soluble factors such as cytokines, and the interaction between carcinoma and stromal cells is strongly associated with tumor malignancy [4]. Chemokines, the member of cytokines, are induced by inflammatory signals such as proinflammatory cytokines, growth factor and pathogenic stimuli and attract leukocytes into tumor tissues [5]. On the other hand, it is well-known that chemokines also regulate tumor growth, invasion, metastasis and angiogenesis through binding to their receptors expressed in tumor cells [6].

C-C motif chemokine ligand (CCL) 5, also known as RANTES, is expressed in various types of stromal cells, such as lymphocytes, macrophages, fibroblasts and adipocytes, and serves as a chemoattractant and recruits immune cells into inflammatory sites. Recently, stromal CCL5 has been implicated in several kinds of human malignancies [7], although some kinds of malignant cells also express CCL5. Previous studies have shown that CCL5 secreted from tumor-associated macrophages (TAMs) promoted progression in prostate, gastric and colorectal cancers [8,9,10]. It has been also demonstrated that cancer-associated fibroblast (CAF)-derived CCL5 has important roles for cisplatin resistance in ovarian cancer [11], and CD4+ cell-derived CCL5 is involved in the growth and immune escape of gastric carcinoma cells [12]. In breast cancer, CCL5 secreted from TAMs has been reported to contribute to tumor malignancy by promoting epithelial–mesenchymal transition (EMT) and aerobic glycolysis [13]. Furthermore, in a previous immunohistochemical study using 40 triple-negative breast cancer (TNBC) tissues, CCL5 immunoreactivity in peritumoral adipocytes was significantly correlated with lymph node metastasis, distant metastases and a worse prognosis in TNBC patients [14].

CCL5 action is mediated through its receptors, C-C chemokine receptor (CCR) 1, CCR3 and CCR5, and carcinoma cells have different expression patterns of these chemokine receptors [6]. Although the roles of CCL5 and its receptors on breast cancer progression have been previously examined in vitro and in vivo by several groups, the results were not always consistent. For instance, it has been reported that the CCL5-CCR5 axis enhances the proliferation of breast carcinoma cells by regulating the energy metabolism [15], while Mañes S [16] demonstrated that CCR5 activity suppresses the progression of breast cancer in a p53-dependent manner. More importantly, the expression of CCL5 and/or its receptors have so far not been well-examined in human breast carcinoma tissues. Therefore, we immunolocalized CCL5, CCR1, 3 and 5 in 111 human breast carcinoma tissues and examined the significance of CCL5 in stromal cells and CCR1, 3, and 5 in tumor cells in order to better understand in more detail the clinical and/or biological significance of the CCL5/receptors axis in breast cancer.

## 2. Results

### 2.1. Expression of CCL5, CCR1, CCR3 and CCR5 in 111 Breast Carcinoma Tissues

**Figure 1:**
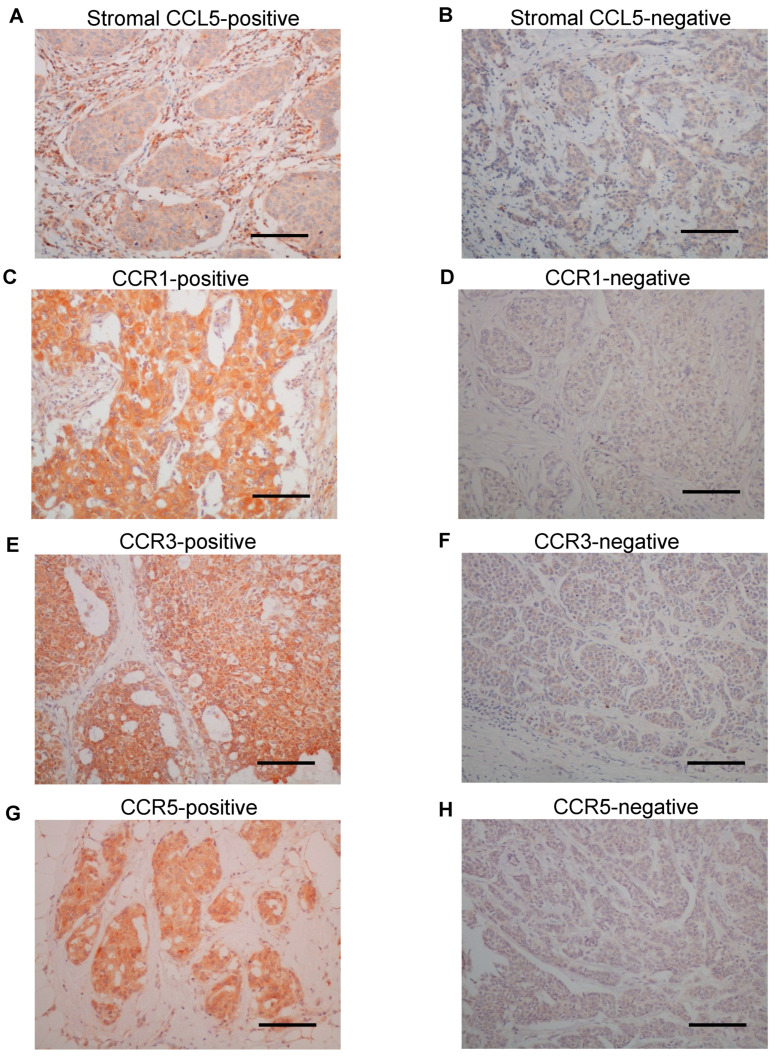
Immunolocalization of C-C chemokine ligand (CCL) 5 and C-C chemokine receptor (CCR) 1, CCR3 and CCR5 in human breast carcinoma tissues. (**A**,**B**) Representative images of the immunoreactivity for CCL5 in the stroma cells. (**C**–**H**) Immunoreactivity for CCR1 (**C**,**D**), CCR3 (**E**,**F**) and CCR5 (**G**,**H**) was detected in the cytoplasm of breast carcinoma cells. (**A**,**C**,**E**,**G**) Positive cases and (**B**,**D**,**F**,**H**) negative cases. Bar = 100 μm, respectively.

Representative images of immunohistochemistry for CCL5, as well as CCR1, 3 and 5, are shown in Figure 1. Immunoreactivity for CCL5 was detected in both stromal cells (Figure 1A,B) and breast carcinoma cells (Figure S1A,B). On the other hand, the immunoreactivity of CCR1 (Figure 1C,D), CCR3 (Figure 1E,F) and CCR5 (Figure 1G,H) were observed in the cytoplasm of breast carcinoma cells. According to the scoring criteria as described in the Materials and Methods, 35% (39 out of 111 case), 19% (21 out of 111 cases), 65% (72 out of 111 cases) and 41% (45 out of 111 cases) were considered positive for CCL5 in stromal cells and CCR1, 3 and 5 in carcinoma cells, respectively.

When we correlated immunoreactivity for CCL5, CCR1, 3 and 5 with the clinicopathological factors of 111 breast carcinoma tissues. As shown in Table 1, CCL5 immunoreactivity was significantly correlated with the histological grade (*p* = 0.0061), microvessel density (MVD) (*p* = 0.040) and CD163-positive macrophage infiltration (*p* = 0.0070), while it tended to be correlated with the Ki67-labeling index (LI) (*p* = 0.090), although the *p*-value did not reach a significant level. On the other hand, CCL5 immunoreactivity was negatively correlated with the estrogen receptor (ER) (*p* = 0.0043) and progesterone receptor (PR) (*p* = 0.0010). Correlations between the clinicopathological factors and CCR1, 3 and 5 immunoreactivity are summarized in Table S1. Briefly, CCR1 immunoreactivity was positively correlated with human epidermal growth factor receptor 2 (HER2) (*p* = 0.025), while no significant correlation was detected between the clinicopathological factors and immunoreactivity for CCR3 and CCR5. We then correlated CCL5 immunoreactivity with clinicopathological factors according to the status of CCR1, 3 and 5, respectively. As shown in Table 2, CCL5 immunoreactivity was not correlated with any clinicopathological factors in the CCR1-positive group (*n* = 21), while it was negatively correlated with pathological T factor (pT) (*p* = 0.037) and lymph node metastasis (*p* = 0.024) in the CCR5-positive group (*n* = 45). In contrast, CCL5 immunoreactivity was positively correlated with the histological grade (*p* = 0.028) and MVD (*p* = 0.044) and tended to be correlated with Ki67 LI (*p* = 0.062) while negatively correlated with the ER (*p* = 0.040) and PR (*p* = 0.0057) in the CCR3-positive group (*n* = 72).

From these findings, we speculated that CCL5 might promote breast cancer progression via CCR3 but not CCR1 or CCR5. To confirm this hypothesis, we divided the patients into two groups—namely, CCR1-negative/CCR3-positive/CCR5-negative and others and compared the clinicopathological features of CCL5 between them (Table 3). In the CCR1-negative/CCR3-positive/CCR5-negative group (*n* = 34), the CCL5 immunoreactivity was significantly correlated with the pT (*p* = 0.017), histological grade (*p* = 0.0015), Ki67 LI (*p* = 0.037) and MVD (*p* = 0.025) and tended to be correlated with lymph node metastasis (*p* = 0.056) while negatively correlated with the ER (*p* = 0.013) and PR (*p* = 0.0009). On the other hand, it was not significantly correlated with any clinicopathological factors in the “others” group (*n* = 77).

### 2.2. Association between Immunoreactivity for CCL5, CCR1 and CCR3 and CCR5 and the Clinical Outcome of 111 Breast Cancer Patients

**Figure 2:**
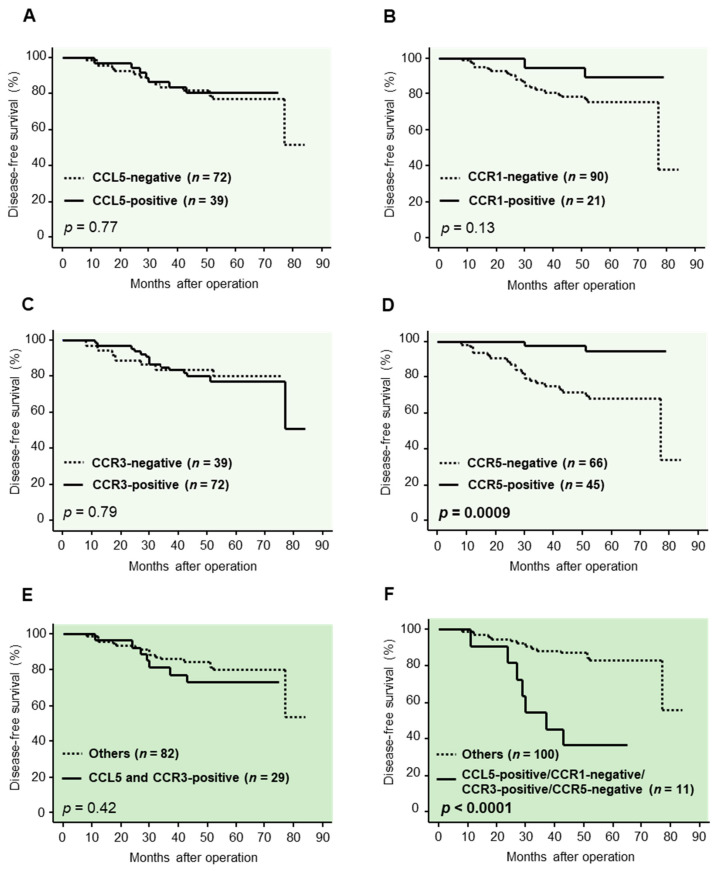
Association between the immunoreactivity for C-C motif chemokine ligand (CCL) 5; C-C chemokine receptor (CCR) 1, CCR3 and CCR5 and the clinical outcome of 111 breast carcer patients. (**A**–**D**) Disease-free survival curve according to the immunoreactivity for CCL5 (**A**), CCR1 (**B**), CCR3 (**C**) and CCR5 (**D**), respectively. (**E**,**F**) Disease-free survival curve in CCL5-positive/CCR3-positive cases and other cases (**E**) and in CCL5-positive/CCR1-negative/CCR3-positive/CCR5-negative cases and other cases (**F**). Kaplan–Meier method was used for generating survival curves, and *p*-values were calculated by log-rank test.

We next examined the prognostic significance of CCL5, as well as its receptors, by evaluating the disease-free survival. As shown in Figure 2A–D, the immunoreactivity for CCL5, CCR1 and 3 was not significantly correlated with a risk of recurrence, while the CCR5 immunoreactivity was significantly correlated with decreased risk of recurrence (*p* = 0.0009).

We then compared the survival curves in the CCL5-positive/CCR3-positive cases and other cases, but no significant correlation was detected (Figure 2E). On the other hand, as shown in Figure 2F, the risk of recurrence was significantly higher in the CCL5-positive/CCR1-negative/CCR3-positive/CCR5-negative cases compared with other patients (*p* < 0.0001).

## 3. Discussion

The tumor microenvironment consists of many stromal cells, as well as carcinoma cells, and stromal cells are known as crucial participants in breast cancer development and therapeutic responses [17]. Chemokines have important roles in the interactions between carcinoma cells and stromal cells in the tumor microenvironment, and CCL5, a member of chemokine family, is expressed in many stromal cells, such as macrophages [8,9,10,13], fibroblasts [11,18,19], lymphocytes [12] and adipocytes [14], in various types of cancers. Furthermore, it has been known that many chemokine receptors are expressed in carcinoma cells and regulate tumor progression [6]. In our present study, we performed immunohistochemistry for CCL5 and its receptors, CCR1, 3 and 5, in 111 invasive breast carcinoma tissues. CCL5 immunoreactivity was markedly detected in stromal cells adjacent to carcinoma cells, as compared to those in normal mammary stroma. Furthermore, the immunoreactivity for CCR1, 3 and 5 was detected in the cytoplasm of carcinoma cells. While chemokine receptors belong to the G protein-coupled receptor superfamily localized on the cell membrane, some of them, including CCR1, 3 and 5, are known to internalize to the cytoplasm following interactions with its ligands [20,21,22], and the cytoplasmic immunoreactivity of CCR1, 3 and 5 may suggest that these receptors are “active” in breast cancer tissues. The CCL5/CCRs axis was therefore considered to have a pivotal role in breast cancer, and we subsequently examined the relationship between CCL5/CCRs and their clinicopathological parameters.

Stromal CCL5 immunoreactivity was significantly correlated with the histological grade and MVD and tended to be correlated with Ki67, a cell proliferation marker, and negatively correlated with the ER and PR and suggested to be associated with tumor growth, invasion and angiogenesis. CCL5 is known to be associated with tumor progression in various cancers, including breast cancer [7], and CCL5 has been reported to regulate the energy metabolism and EMT in breast carcinoma cells, contributing to breast cancer metastasis [13]. Furthermore, in the previous immunohistochemical study, CCL5 expression in peritumoral adipocytes was correlated with the highly aggressive phenotype and a worse prognosis in TNBC patients [14]. Taken together, with these previous studies and our findings, stromal CCL5 may contribute to tumor progression, such as cell proliferation, invasion and angiogenesis, in breast carcinoma tissues. Furthermore, CCL5 immunoreactivity was significantly correlated with macrophage infiltration, and previous studies have been also shown that macrophage infiltration contributes to tumor growth, metastasis, angiogenesis and drug resistance in breast cancers [23,24,25,26]. Considering that CCL5 is known as a chemoattractant for macrophages [7], CCL5 might promote tumor progression partly by recruiting macrophages to the tumor area.

CCR1, 3 and 5 are known as the receptors of CCL5, and we then evaluated them in breast carcinoma tissues and examined the association between CCL5 immunoreactivity in stromal cells and clinicopathological factors according to the status of CCR1, 3 and 5 in carcinoma cells. CCL5 immunoreactivity was correlated with the aggressive phenotype of breast carcinomas only in the CCR3-positive group, and we speculated that the CCL5-CCR3 interaction might have important role in tumor progression. In the CCR1-negative/CCR3-positive/CCR5-negative group, CCL5 immunoreactivity was significantly correlated with the pT, histological grade, Ki67 LI and MVD and tended to be correlated with lymph node metastasis while negatively correlated with the ER and PR. CCR3 has been reported to promote tumor progression in some kinds of carcinomas, showing agreement with our findings. For instance, it has been demonstrated that the CCL11-CCR3 interaction promotes the cell survival of anaplastic large cell lymphoma cells and the invasion of prostate cancer cells by the activation of extracellular signal-regulated kinase (ERK) [27,28], and the CCL7-CCR3 axis contributes to the metastasis of colon cancer cells by regulating the ERK- Jun amino-terminal kinases (JNK) signaling pathways [29]. Taken together with these previous studies and our findings, CCL5 secreted by stromal cells may contribute to tumor progression by activating CCR3 expressed in carcinoma cells and regulating downstream pathways such as ERK signaling in breast carcinoma tissues.

In contrast, CCL5 immunoreactivity was negatively correlated with pT and lymph node metastasis in the CCR5-positive group. CCR5 has been reported to regulate p53 transcriptional activity in breast carcinoma cells and suppress the progression of breast cancer in a p53-dependent manner by in vitro and in vivo experiments [16], and the authors also found out that the breast cancer patients with the nonfunctional CCR5∆32 allele showed shorter disease-free survivals compared to those with the wild type. These previous findings are in agreement with our present study, and the CCL5/CCR5 axis may be associated with the less aggressive phenotype of breast cancer in contrast to CCR3. However, it is also true that the CCL5-CCR5 interaction has also been reported to promote breast cancer proliferation by regulating metabolic events [15] and mTOR activation [30]. CCR5 may therefore promote proliferation and invasion in the relatively less aggressive type of breast carcinomas, but it needs to be further examined using a larger sample size of human breast carcinoma tissues and several different types of breast cancer models (cell lines and/or mice models).

We further examined whether CCL5 and CCR1, 3 and 5 served as prognostic factors in breast cancers. In the present study, CCL5 immunoreactivity itself was not significantly correlated with a risk of recurrence in all the cases examined. Among the receptors, only CCR5 was correlated with a longer disease-free survival, and CCR1 and CCR3 were not correlated with disease-free survival at all. Furthermore, the survival curve of the patients with CCL5/CCR3 double-positive breast carcinomas was almost same as the others. However, it should be noted that CCL5 might exert different effects on breast cancer according to the receptors, as described above, and the receptors, the significance of which are different from each other, are co-expressed in the same cells or tissues. It is therefore speculated that the prognostic role of CCR3 is weakened by the co-expression with CCR5. Actually, the risk of recurrence was significantly higher in patients with breast carcinomas positive for CCL5 and CCR3 but negative for CCR1 and CCR5. This finding suggested that the CCL5/CCR3 axis especially contributes to the recurrence of breast cancer and is considered as an important target to improve the treatment of breast cancers. On the other hand, it has been reported that a higher expression of CCR3 mRNA was correlated with an improved relapse-free survival of breast cancer patients [31]. However, it is not necessarily inconsistent with the present study, because the method to evaluate CCR3 was different (public database in the previous report, while immunohistochemistry in the present study). In addition, in the previous study, CCR3 was correlated with an improved relapse-free survival in only the luminal type, but not in other types, of breast cancer. It might be challenging to clarify the differential prognostic role of the immunohistochemical CCR3 status using a larger sample size of breast cancer specimens.

**Figure 3:**
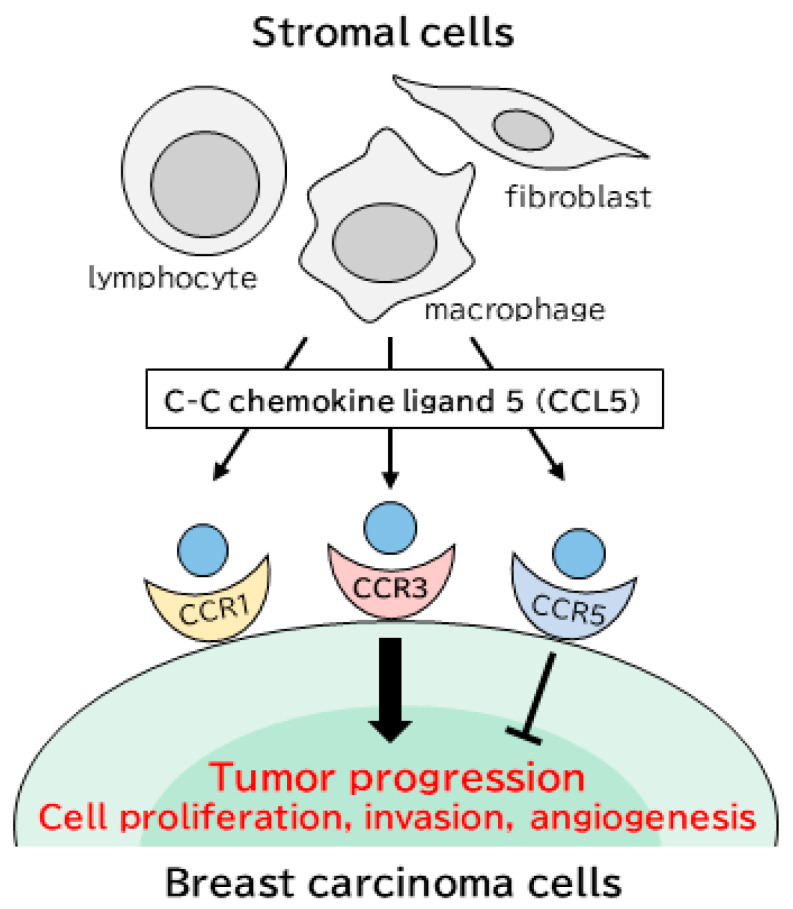
The tumor microenvironment consists of various stromal cells, as well as carcinoma cells. C-C motif chemokine ligand (CCL) 5 is secreted from many stromal cells, such as macrophages, fibroblasts and lymphocytes, and CCL5 action is mediated through its receptors: C-C chemokine receptor (CCR) 1, 3 and 5. Stromal CCL5 contributes to breast cancer progression and a worse prognosis by interacting with CCR3 in tumor cells, while CCL5 is associated with tumor suppression binding to CCR5.

In summary, we immunolocalized CCL5 and CCR1, 3 and 5 in breast carcinoma tissues and demonstrated that the expression of CCL5 in stromal cells was significantly correlated with tumor progression in a paracrine manner via CCR3 expressed in breast carcinoma cells (Figure 3). These findings will be helpful for better understanding the clinical and biological significances of the CCL5/receptors axis in the breast cancer microenvironment, and the CCL5-CCR3 axis may be an important target to improve the prognosis of breast cancer patients.

## 4. Materials and Methods

### 4.1. Patients and Tissues

Specimens (111) of invasive breast carcinoma tissues were obtained from female patients who underwent surgical treatment from 2007 to 2008 in the Department of Surgery, Tohoku University Hospital, Sendai, Japan. All specimens were fixed by 10% formalin neutral buffer solution and embedded in paraffin. Disease-free survival was defined as the period from the date of surgery to the first locoregional recurrence or distant metastasis, and the mean follow-up time after surgery was 59 months (range 3–84).

This research protocol was approved by the Ethics Committee at the Tohoku University Graduate School of Medicine (approval No. 2016-1-697; 16 February 2017 and No. 2019-1-219; 11 July 2019).

### 4.2. Immunohistochemistry

In Table S2, the information of antibodies was listed. Immunohistochemistry for CCL5, CCR1, CCR3, CCR5, CD31, Ki67 and CD163 was carried out using a Histofine kit (Nichirei Bio, Inc., Tokyo, Japan), as described previously [32,33,34]. The antigen–antibody reaction was visualized with 3,3′- diaminobenzidine (DAB) and counterstained with hematoxylin. Immunohistochemistry for ER and PR was carried out using the Ventana Benchmark XT system (Roche Diagnostics Japan, Tokyo, Japan) and, for HER2, was carried out with the HercepTest (Dako, Carpinteria, CA, USA).

### 4.3. Scoring of Immunoreactivity

Immunoreactivity for CCL5 was detected in the cytoplasm of both the stroma and breast carcinoma cells. We put more attention on the role of CCL5 secreted from breast cancer stromal cells in this study. Regarding the evaluation of stromal CCL5 immunoreactivity, it was considered positive when the cases had an immunoreactivity of more than 30% of the stromal cells, although there were no standardized criteria for the CCL5 staining evaluation in the stroma of carcinoma tissues [13]. Immunoreactivity for CCR1, 3 and 5 was detected in the cytoplasm of breast carcinoma cells, and the cases with immunoreactivity of more than 10% of the carcinoma cells were considered positive for them. CD31 immunoreactivity was detected in the vascular endothelium (Figure S1C,D). Microvessels were then counted at ×200 magnification with a Nikon Eclipse 80i microscope (Nikon, Tokyo, Japan) in five fields within the hotspots, according to a previous report [35], and MVD was defined as the average of the microvessels in five fields. CD163 immunoreactivity was detected in the cell membranes of the macrophages (Figure S1E,F), and the number of CD163-positive macrophages was categorized into 4 groups (0: no focal areas, 1: small scattered focal areas, 2: numerous larger focal areas and 3: very numerous large focal areas), according to a previous report [36] and, finally, dichotomized (0 to 1: low infiltration and 2 to 3: high infiltration). Immunoreactivity for ER, PR and Ki67 (Figure S1G,H) were detected in the nuclei of breast carcinoma cells. By evaluating more than 1000 cells, the percentage of positive cells (labeling index; LI) was calculated. Cases with LI of more than 1% were determined positive for ER and PR, according to a previous report [37]. HER2 immunoreactivity was determined according to the grading system proposed in the HercepTest (Dako).

### 4.4. Statistical Analyses

Statistical analyses were carried out with JMP Pro 14.0.0 (SAS Institute, Cary, NC, USA). χ^2^ test or Mann–Whitney *U* test were applied for the correlations between CCL5, CCR1, 3 and 5 and the clinicopathological factors. Disease-free survival curves were generated using the Kaplan–Meier method, and a log-rank test was applied for evaluating the statistical significance. *p* < 0.05 was considered significant in this study.

## Figures and Tables

**Table 1:** Association between C-C motif chemokine ligand 5 (CCL5) and the clinicopathological factors in 111 breast carcinomas.

Clinicopathological Factors	CCL5 Status		*p*-Value
	Negative (*n* = 72)	Positive (*n* = 39)	
Age *	56 (29–82)	56 (27–87)	0.40
Menopause			
Premenopausal	29	12	0.32
Postmenopausal	43	27	
Stage			
I	41	22	0.56
II	17	12	
III	14	5	
Pathological T Factor			
pT1	48	25	0.79
pT2-4	24	14	
Lymph Node Metastasis			
Negative	49	26	0.88
Positive	23	13	
Histological Grade			
1 (well)	32	11	**0.0061**
2 (intermediate)	31	13	
3 (poor)	9	15	
ER			
Negative	8	13	**0.0043**
Positive	64	26	
PR			
Negative	15	20	**0.0010**
Positive	57	19	
HER2			
Negative	63	33	0.67
Positive	9	6	
Ki67 LI (%) *	9.5 (1–60)	16 (1–49)	*0.090*
MVD *	24.5 (3–101)	32 (7–106)	**0.040**
Macrophage infiltration			
Low	55	20	**0.0070**
High	17	19	

* Data were presented as median (minimum–max). All other values represent the number of cases. *p* < 0.05 was considered significant and described in boldface, and 0.05 ≤ *p* < 0.1 was described in italic. ER: estrogen receptor, HER2: human epidermal growth factor receptor 2, LI: labeling index, MVD: microvessel density and PR: progesterone receptor.

**Table 2:** Association between CCL5 and the clinicopathological factors according to the C-C chemokine receptor 1 (CCR1), CCR3 and CCR5 status in breast carcinomas.

Clinicopathological Factors	CCR1-Positive Group			CCR3-Positive Group			CCR5-Positive Group		
	CCL5 Status		*p*-Value	CCL5 Status		*p*-Value	CCL5 Status		*p*-Value
	Negative (*n* = 11)	Positive (*n* = 10)		Negative (*n* = 43)	Positive (*n* = 29)		Negative (*n* = 25)	Positive (*n* = 20)	
Stage									
I	5	5	0.36	24	17	0.73	13	16	0.13
II	4	5		10	8		7	3	
III	2	0		9	4		5	1	
Pathological T Factor									
pT1	6	8	0.22	27	20	0.60	14	17	**0.037**
pT2-4	5	2		16	9		11	3	
Lymph Node Metastasis									
Negative	7	5	0.53	30	19	0.70	15	18	**0.024**
Positive	4	5		13	10		10	2	
Histological Grade									
1 (well)	4	4	0.14	15	8	**0.028**	11	9	0.92
2 (intermediate)	5	1		22	9		10	7	
3 (poor)	2	5		6	12		4	4	
ER									
Negative	2	3	0.53	6	10	**0.040**	4	7	0.14
Positive	9	7		37	19		21	13	
PR									
Negative	3	5	0.28	10	16	**0.0057**	4	8	*0.070*
Positive	8	5		33	13		21	12	
HER2									
Negative	8	7	0.89	36	24	0.91	20	17	0.66
Positive	3	3		7	5		5	3	
Ki67 LI (%) *	16 (1–30)	19 (1–37)	0.60	13 (1–53)	18 (1–44)	*0.062*	12 (1–53)	13.5 (1–47)	0.73
MVD *	28 (13–101)	30.5 (18–82)	0.84	23 (6–101)	36 (7–106)	**0.044**	28 (10–101)	27 (7–82)	0.62

* Data were presented as median (minimum–max). All other values represent the number of cases. *p* < 0.05 was considered significant and described in boldface, and 0.05 ≤ *p* < 0.1 was described in italic. ER: estrogen receptor, HER2: human epidermal growth factor receptor 2, LI: labeling index, MVD: microvessel density and PR: progesterone receptor.

**Table 3:** Association between CCL5 and the clinicopathological factors in CCR1-negative/CCR3-positive/CCR5-negative breast carcinoma cases and other cases.

Clinicopathological Factors	CCR1-Negative/CCR3-Positive/ CCR5-Negative Cases			Others		
	CCL5 Status		*p*-Value	CCL5 Status		*p*-Value
	Negative (*n* = 23)	Positive (*n* = 11)		Negative (*n* = 49)	Positive (*n* = 28)	
Stage						
I	15	4	0.27	26	18	0.30
II	4	4		13	8	
III	4	3		10	2	
Pathological T Factor						
pT1	18	4	**0.0017**	30	21	0.22
pT2-4	5	7		19	7	
Lymph Node Metastasis						
Negative	18	5	*0.056*	31	21	0.29
Positive	5	6		18	7	
Histological Grade						
1 (well)	8	0	**0.0015**	24	11	0.35
2 (intermediate)	12	3		19	10	
3 (poor)	3	8		6	7	
ER						
Negative	2	5	**0.013**	6	8	*0.074*
Positive	21	6		43	20	
PR						
Negative	5	9	**0.0009**	10	11	*0.074*
Positive	18	2		39	17	
HER2						
Negative	20	10	0.74	43	23	0.50
Positive	3	1		6	5	
Ki67 LI (%) *	13 (1–49)	22 (3–44)	**0.037**	9 (160)	12 (1–49)	0.44
MVD *	22 (6–52)	40 (14–106)	**0.025**	27 (3–101)	28.5 (7–82)	0.38

*; Data were presented as median (minimum–max). All other values represent the number of cases. *p* < 0.05 was considered significant and described in boldface, and 0.05 ≤ *p* < 0.1 was described in italic. ER: estrogen receptor, HER2: human epidermal growth factor receptor 2, LI: labeling index, MVD: microvessel density and, PR: progesterone receptor.

## Data Availability

All data and materials presented in this article and in the supplementary information are available from the corresponding author upon reasonable request.

## Supplementary Materials

The following are available online at https://www.mdpi.com/1422-0067/22/4/1918/s1. Figure S1: Immunolocalization of C-C chemokine ligand (CCL) 5, CD31, CD163 and Ki67 in human breast carcinoma tissues. Table S1: Association between CCR1, CCR3 and CCR5 and the clinicopathological factors in 111 breast carcinomas. Table S2: Information on the antibody used in this study.
